# A case report of an endometriosis cyst at cesarean scar defect and review of literature

**DOI:** 10.1186/s12884-022-05311-9

**Published:** 2022-12-21

**Authors:** Ruibo Xu, Xinlei Xia, Ying Liu, Xiaoli Du, Zengfang Hao, Lili Wang, Jiexian Du

**Affiliations:** 1 Department of Gynecology, The Second Hospital of Hebei Medical University, Shijiazhuang, 050000 Hebei China; 2 Department of Gynecology, Traditional Chinese Medicine Hospital of Shijiazhuang, Shijiazhuang, 050000 Hebei China; 3 Department of Pathology, The Second Hospital of Hebei Medical University, Shijiazhuang, 050000 Hebei China; 4 Department of Gynecology, Eastern Hospital, Second Hospital of Hebei Medical University, No. 80, Huanghe Avenue, Shijiazhuang, Hebei China

**Keywords:** Pelvic mass, Cesarean scar defect, Endometriosis, Endometriosis cyst, Case report

## Abstract

**Background:**

Cesarean scar defect (CSD) presents as a cystic defect that connects the uterine cavity at the site of the previous cesarean section (CS). Endometriosis refers to the discovery of endometrial glands and stroma outside the uterine cavity. Cases of endometriosis cysts at CSD have not been reported.

**Case presentation:**

In this article, we will present a patient with an endometriosis cyst at CSD with symptoms of a prolonged menstrual cycle, periods without cyclic abdominal pain, and a history of cesarean delivery. The gynecologic ultrasound showed a CSD and a mixed mass in the right front of the uterus. After about 1 month, the tumor grew from a diameter of 4.75 cm to 8.06 × 6.23 × 3.66 cm. The patient eventually had an operation, which revealed a mass protruding from the incision in the anterior uterine wall, which was attached to the anterior uterine wall by a thin tip with a smooth surface. Intraoperative rapid cytopathology suggested that endometrial glands were seen within the smooth muscle tissue, similar to endometriosis. Subsequently, the patient underwent resection of the endometriotic cyst. Final paraffin pathology showed smooth muscle with visible endometrial glands and old hemorrhage, and a one-year follow-up showed no recurrence of endometriosis cysts at CSD.

**Conclusions:**

Endometriosis cysts at CSD are very rare. The clinical symptoms may be less obvious, and the diagnosis relies mainly on the patient’s previous surgical history and imaging. A finding of a pelvic mass in the location of the CSD, with or without symptoms of menstrual changes and intermittent abdominal pain, should be considered an endometriotic cyst at CSD. Surgical treatment is a good choice for this disease. Further studies are needed regarding the etiological mechanism of this case and why the mass enlarged rapidly in one mouth.

## Introduction

Morris first proposed cesarean scar defect (CSD) in 1995, also referred to as cesarean scar diverticulum, isthmocele, uterine scar defect, uterine diverticulum, uterine niche, and uterine scar defect or sacculation [1]. Due to the significant increase in the cesarean section (CS) rate, the incidence of CSD increased to 61% after one CS and up to 100% after at least three times CSs [2, 3]. Notably, CSD presents as a cystic defect connecting the uterine cavity at the site of the previous cesarean section (CS), resulting from incomplete scar healing at the anterior uterine isthmus [3, 4]. Consequently, CSD can lead to numerous gynecological and obstetric disorders, such as abnormal uterine bleeding, punctate bleeding after menstruation, uterine dehiscence, uterine rupture, cesarean scar pregnancy, and morbidly adherent placenta [5]. Endometriosis refers to the discovery of endometrial glands and stroma outside the uterine cavity [6]. Notably, scar endometriosis can often result in the discovery of endometriosis tissue at the surgical incision site after obstetric or gynecological surgeries [7]. Due to scar endometriosis often presenting as cysts at the scar, which also presents some similarity to the content of this case, investigations of scar endometriosis may provide added guidance for the diagnosis and management of this case.

This article will present a patient with an endometriosis cyst at CSD, with symptoms of prolonged menstrual cycle and periods, without periodic abdominal pain, and a history of CS. To our knowledge, no such cases have been reported.

## Case presentation

A 40-year-old woman complained of menstrual irregularity lasting for 2 years, and the patient reported menstrual periods lasting 3 to 15 days and cycles of 15-60 days. The patient’s menstrual volume varies; at most, it can be 4 times the average. However, the patient has never had periodic abdominal pain. The patient does have a history of two previous CS, and the last CS occurred 12 years ago. No other history beyond the cesarean was noted, and there is no knowledge of any familial genetic disease.

**Fig. 1:**
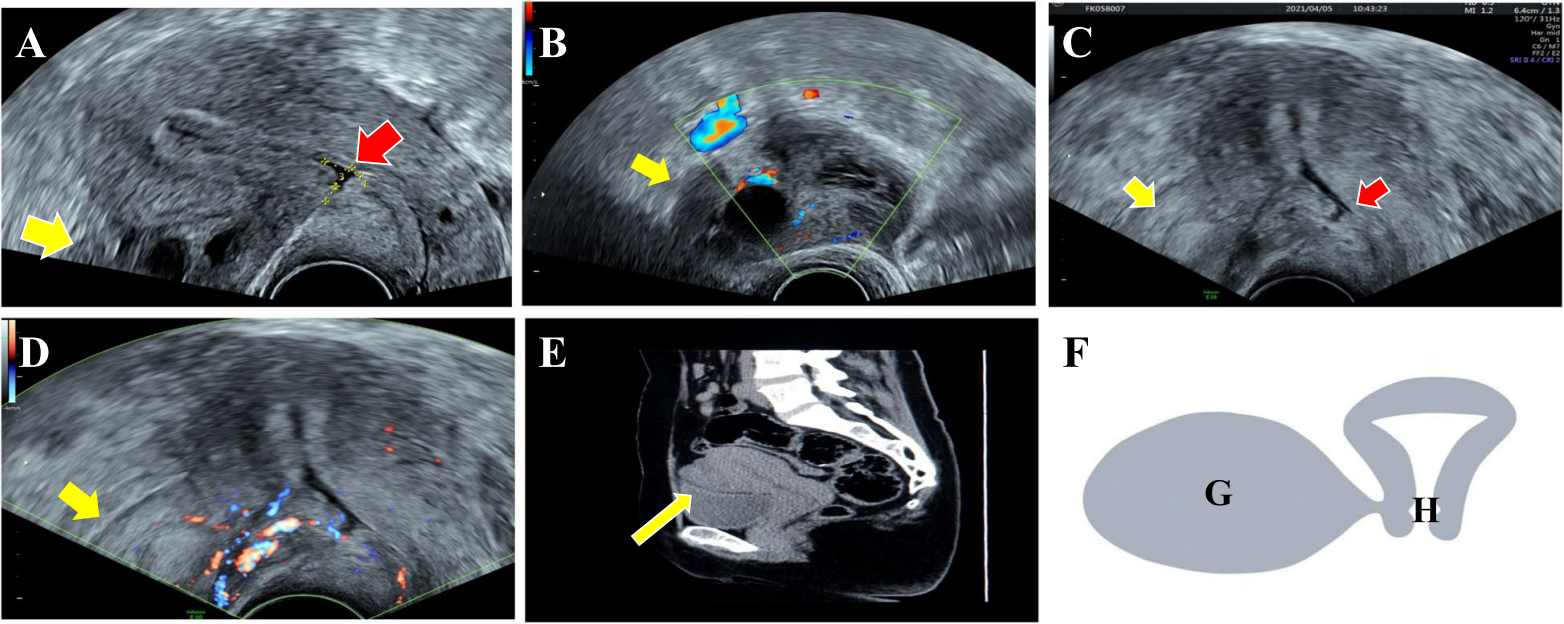
Gynecological ultrasound image **A**-**D** The red arrow indicates cesarean scar diverticulum, and the yellow arrow indicates mass. **A** A diameter of about 4.75 cm mass in the cesarean scar of the uterus. **B** Color Doppler ultrasound: visible a little blood flow signal. **C** A diameter of about 8.06 × 6.23 × 3.66 cm mass in the cesarean scar of the uterus. **D** Color Doppler ultrasound: visible blood flow signal. **E** Pelvic CT: the yellow arrow indicates mass, and the spindle shape is the transverse section of the uterus. **F** Schematic diagram of mass location: **G** is mass, **H** is cesarean scar diverticulum, and **G** and **H** are not connected

The gynecological ultrasound of the patient showed that the incision at the anterior wall of the uterus had a triangular defect with an accessible base of 0.76 cm and a height of 0.55 cm. A dark area with an inner diameter of about 4.75 cm could be seen in the pelvis, and a grid-like high echo could be seen in the dark area. Notably, Color Doppler ultrasound showed no significant abnormal blood flow signal (Fig. 1A, B). Alpha-fetoprotein, carcinoembryonic antigen, carbohydrate antigen 19.9, carbohydrate antigen 125 (CA125), and human epididymis protein 4 were within normal limits.

One month later, the patient returned, and gynecological ultrasound revealed a mixed mass measuring approximately 8.06 × 6.23 × 3.66 cm in the right anterior part of the uterus, with hypoechoic, anechoic, and hyperechoic areas, closely associated with the uterine body incision. Furthermore, Color Doppler ultrasound suggested a slight enrichment of blood flow within the mass (Fig. 1C, D). The pelvic CT result showed a spindle shape in the transverse section of the uterus. We observed a solid cystic mass of approximately 5.26 × 4.24 cm with CT values of approximately 17-50 Hu (Fig. 1E, F).

**Fig. 2:**
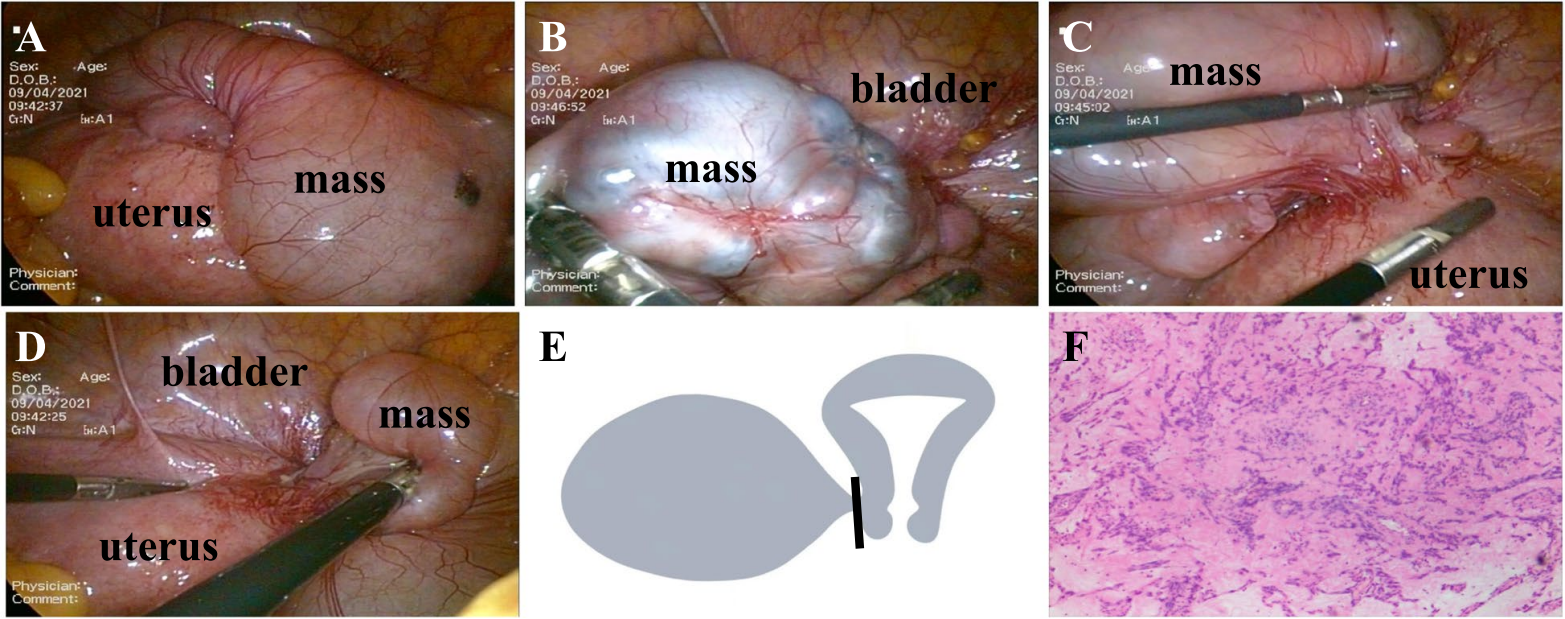
Laparoscopic images(**A**-**D**). **A** The mass is located above the uterus. **B** Some brown liquid can be seen in the mass. **C**&**D** The mass is connected with the cesarean incision scar of the anterior wall of the uterus by a pedicle. **E** Schematic diagram of operation mode: completely cut off the mass at its pedicle. **F** Paraffin pathological results: endometrial glands are visible

Finally, the patient underwent laparoscopic surgery. This procedure found a cystic mass at the incision of the anterior uterine wall with a thin tip attached to the anterior uterine wall (Fig. 2A, B, C, D). The cyst was removed entirely (Fig. 2E) and sent for intraoperative snap-frozen pathology, suggesting endometrium-like glands and minor bleeding visible in the smooth muscle tissue, which was inclined to endometriosis. The gross specimen was approximately 8 × 6 × 4 cm, smooth, with multiple visible cystic cavities, some with clear yellow fluid, some with dark brown fluid, some with thicker brown fluid, and smooth cystic walls. The final paraffin pathology showed endometrial glands and old hemorrhage within the smooth muscle (Fig. 2F). After the operation, the patient recovered well and was very satisfied with the treatment result. Furthermore, a one-year follow-up showed no recurrence of endometriosis cysts at the CSD.

## Discussion

Any myometrium defect in the lower part of the uterus after uterine surgery may be considered CSD [1]. Most of the scar endometriosis reported in the case occurred solely at the surgical incision site [7–9]. This case of endometriosis cyst at CSD has not been reported.

There is no definite conclusion about the occurrence of CSD, which is mainly related to poor wound healing [2, 5]. Some studies have identified independent risk factors for CSD, including a history of gestational diabetes, previous CS, and a late maternal body mass index [2]. In addition, another study found that the occurrence of CSD was significantly associated with recurrent CS, premature rupture of membranes, short operative time, and CS cervical dilatation [10]. Furthermore, a cohort study noted that perioperative infection and hypercoagulable state should be considered risk factors for CSD [5]. However, the exact etiology of CSD still needs further investigation. Likewise, the mechanism of scar endometriosis is not completely clear. It is generally accepted that endometrial tissue survives directly at the surgical wound site during surgery and then is impacted by hormonal and growth factors to form endometriosis [7, 11]. Another hypothesis suggests that metaplasia of coelomic epithelium may also contribute to disease development [9]. Notably, with the discovery of endometriosis far from the pelvis, some scholars believe that endometrial tissue may reach the wound through the lymphatic or vascular pathways [7].

The possible reason for the formation of an endometriosis cyst at CSD described in this paper is that during CS, the endometrium leaves the uterine cavity and reaches the plasma surface of the uterus. Under the action of hormones and growth factors, the ectopic endometrium affects the healing of the cesarean incision. The two interact with each other, thus forming the endometriosis cyst at CSD. In this article’s case of the endometriosis cyst at CSD, the diameter of the mass increases from 4 cm to 8 cm in 1 month. In addition to the mass enlargement of the ectopic endometrium under the periodic action of hormones, there may also be the influence of CSD. CSD has scar uterine incision myometrium discontinuity. Menstruation may pass through the defective uterine base, directly increasing the tumor’s size and increasing the tumor rapidly in a short time. Or the environment of CSD is more conducive to the growth of peculiar smell endometrium, and the activity of ectopic endometrium is high, leading to the above situation. Alternatively, the interaction of two factors may lead to the rapid growth of a tumor.

In summary of the patient diagnosis and treatment, the diagnosis of endometriosis at CSD is mainly based on the patient’s previous history of one or more CSs, clinical manifestations, and imaging examinations to confirm the diagnosis.

Most CSD patients are asymptomatic, but 28.9-82% will show abnormal and/or post-menstrual bleeding (AUB) [1]. Lumps and intermittent pain are the most common symptoms of scar endometriosis [9]. The patient described in the article had only menstrual changes and no periodic abdominal pain. Therefore, when a patient finds a pelvic mass in the location of the CSD scar, with or without symptoms of menstrual changes and intermittent abdominal pain, it should be considered an endometriotic cyst at CSD.

The auxiliary examination for endometriosis cysts at CSD mainly involves imaging examinations. Ultrasound, CT, and MRI can assist in diagnosing, but the final diagnosis requires histological diagnosis [7, 12]. On ultrasound, the defect may present as an anechoic, triangular shape defect with an apex pointing anteriorly and located at the anterior isthmus [12]. Furthermore, endometriosis at CSD may present ultrasonically as a cystic, solid, or heterogeneous mass in the location of a CSD [7]. In the case described here, no significant blood flow signal was found in the mass on the first Color Doppler ultrasound and only identified with the second ultrasound examination 1 month later. The reason may be that in some cases, color Doppler ultrasound in endometriosis at CSD may not show a blood flow signal or that endometriosis at CSD is affected by CSD and some other factors that cause rapid formation within 1 month. Magnetic resonance imaging (MRI) is not operator reliant. It can help observe the size of CSD and measure the thickness of the residual myometrium thickness (RMT). However, for RMT measurements, the differences between TVS and MRI were not statistically significant [13]. Importantly, scar endometriosis usually presents as a confined solid or mixed mass on CT. Compared to MRI, hemorrhagic foci in the lesions can be observed with CT [7]. If MRI shows a blood component in the mass, it is suggestive of endometriosis [9]. In addition to imaging tools, several other complementary tests are used to diagnose endometriosis. Fine-needle aspiration (FNA) is a simple, non-invasive, and easy-to-implement preoperative diagnosis method, which is very important for excluding the malignant transformation of tumors before an operation [14]. Furthermore, the overexpression of CA125 is considered an important marker for the diagnosis of endometriosis. However, negative CA125 does not rule out endometriosis or malignant tumors [15].

There is no current gold-standard treatment for CSD, and common treatment modalities include hormonal medication, surgical repair, or even hysterectomy for definitive treatment [1]. For CSD patients with symptoms of AUB, hormonal drug treatment is an effective option [16]. Surgical treatment of CSD remains controversial and should be offered to women who are symptomatic or asymptomatic but desire to have children [13, 16]. Hysteroscopy has the advantages of a short operation time, less bleeding, short hospital stay, and low hospital cost [17]. However, hysteroscopy can only be performed by excision and cannot be repaired. Therefore, it is not an ideal option for women with RMT < 3 mm who desire to become pregnant [13]. In contrast, transvaginal repair and laparoscopic repair are both viable repair methods. Vaginal repair is simple and different and applies to all patients [18]. Before the procedure of laparoscopic surgery, identifying the location of defects is a considerable difficulty. Therefore, some researchers recommend using laparoscopy and hysteroscopy combined with surgery. Through hysteroscopy, defects can be accurately located under the laparoscopy [19]. Laparoscopic surgery, while treating CSD, can explore other possible causes of infertility and pain and can be easily corrected at the uterine flexion [13].

Current treatment of scar endometriosis includes pharmacological management and direct surgical excision therapy [14]. Notably, medication can alleviate the patient’s symptoms and inhibit the lesion’s amplification by hormones [7]. Furthermore, combining oral contraceptives, progestogens, and hormone suppression therapy with gonadotropin-releasing hormone (GnRH) analogs are useful [14]. Of the various options, surgical excision is the most effective method for diagnosing and treating scar endometriosis, and the surgical extent should include a clear edge of at least 1 cm from the tissue [7]. Malignant tumors have been reported in scar endometriosis, and although this transformation is very rare, surgical excision of the diseased tissue is important and necessary [20]. In our case, the endometriotic lesion was completely resected intraoperatively, and the patient was followed up for 1 year with no significant recurrence.

## Conclusions

Endometriotic cysts at CSDs are rare, and no similar cases have been reported. Additionally, the clinical symptoms of endometriotic cysts at CSDs may be less pronounced. The diagnosis relies heavily on the patient’s previous surgical history and imaging. Ultrasound and MRI are good imaging tests to aid in the diagnosis. Patients with a history of gynecologic or obstetric surgery, the presence or absence of menstrual changes and/or periodic abdominal pain, and the finding of a pelvic mass should be considered for endometriotic cysts at CSD. Surgical treatment seems to be a promising treatment option for the patient. It is also important to note that the mass may grow rapidly in the short term and show abnormal blood flow signals on gynecologic ultrasound. However, this does not imply malignancy. It is particularly regrettable that this article only describes the disease but does not provide an in-depth study of the causes of its formation and some specific symptoms. Therefore, studies are needed regarding the causes of endometriotic cysts at CSD, the rapid increase in the mass size within a month, and whether there is an interaction between CSD and endometriotic cysts.

## Data Availability

All data generated or analysed during this study are included in this published article.

## Abbreviations

CSD: Cesarean scar defect
CS: Cesarean section
AWE: Abdominal wall endometriosis
CT: Computer tomography
AUB: Abnormal uterine bleeding
MRI: Magnetic resonance imaging
RMT: Residual myometrium thickness
TVS: Transvaginal sonography
FNA: Fine-needle aspiration
CA125: Carbohydrate antigen 125
GnRH: Gonadotropin-releasing hormone
